# Inhibition of Myostatin Signaling through Notch Activation following Acute Resistance Exercise

**DOI:** 10.1371/journal.pone.0068743

**Published:** 2013-07-02

**Authors:** Matthew G. MacKenzie, David Lee Hamilton, Mark Pepin, Amy Patton, Keith Baar

**Affiliations:** 1 Division of Molecular Physiology, University of Dundee, Dundee, Scotland, United Kingdom; 2 Health and Exercise Sciences Research Group, School of Sport, University of Stirling, Stirling, United Kingdom; 3 Biomedical Engineering, University of California Davis, Davis, California, United States of America; 4 Department of Neurobiology, Physiology, and Behavior, University of California Davis, Davis, California, United States of America; 5 Department of Physiology and Membrane Biology, University of California Davis, Davis, California, United States of America; University of Las Palmas de Gran Canaria, Spain

## Abstract

Myostatin is a TGFβ family member and negative regulator of muscle size. Due to the complexity of the molecular pathway between myostatin mRNA/protein and changes in transcription, it has been difficult to understand whether myostatin plays a role in resistance exercise-induced skeletal muscle hypertrophy. To circumvent this problem, we determined the expression of a unique myostatin target gene, Mighty, following resistance exercise. Mighty mRNA increased by 6 h (82.9±24.21%) and remained high out to 48 h (56.5±19.67%) after resistance exercise. Further examination of the soleus, plantaris and tibialis anterior muscles showed that the change in Mighty mRNA at 6 h correlated with the increase in muscle size associated with this protocol (R^2^ = 0.9996). The increase in Mighty mRNA occurred both independent of Smad2 phosphorylation and in spite of an increase in myostatin mRNA (341.8±147.14% at 3 h). The myostatin inhibitor SKI remained unchanged. However, activated Notch, another potential inhibitor of TGFβ signaling, increased immediately following resistance exercise (83±11.2%) and stayed elevated out to 6 h (78±16.6%). Electroportion of the Notch intracellular domain into the tibialis anterior resulted in an increase in Mighty mRNA (63±13.4%) that was equivalent to the canonical Notch target HES-1 (94.4±7.32%). These data suggest that acute resistance exercise decreases myostatin signaling through the activation of the TGFβ inhibitor Notch resulting in a decrease in myostatin transcriptional activity that correlates well with muscle hypertrophy.

## Introduction

Myostatin, or growth and differentiation factor (GDF-8), is a member of the transforming growth factor (TGF) β superfamily of proteins. Canonically, myostatin association with the activin IIB receptor (ActRIIB) increases Smad2/3-mediated transcription and represses muscle growth [1]. Interfering with the myostatin pathway at any point leads to an increase in muscle size through a poorly understood mechanism. Substantial hypertrophy occurs when myostatin is decreased genetically [2], immunologically using myostatin-specific antibodies [3] and by interfering with the activation of the activin IIB receptor (ActRIIB) either with the myostatin propeptide or the circulating inhibitor follistatin [1], [4], . Additionally, impairing downstream myostatin signaling by increasing SKI, a repressor of Smads, also induces substantial hypertrophy [7], [8]. The fact that so many constituents of this pathway can induce muscle hypertrophy suggests myostatin plays a central role in the regulation of muscle mass by resistance exercise. However, due to the complexity of measuring myostatin activity, this relationship remains unclear.

Even though evidence exists that myostatin is transcriptionally downregulated by resistance exercise [9], [10], there is no correlation between the downregulation of myostatin and muscle growth [11]. This is in stark contrast to components of the mTORC1 pathway that show a tight correlation between their activation following resistance exercise and increases in muscle mass and strength following training [12], [13]. However, only limited evidence using a small number of biomarkers of myostatin activity in response to resistance training exists [14]. Additionaly, when analyzing the potential importance of myostatin signaling in response to resistance exercise no one has effectively measured all of the different aspects of the pathway (i.e. myostatin, propeptide, follistatin, SKI/Sno, etc.) simultaneously to get a true measure of myostatin activity. Marshall et al. [15] have described a direct transcriptional target of myostatin termed Mighty that might aide in the characterization of myostatin activation following resistance exercise. Mighty expression is decreased by myostatin in a dose-dependent manner [15]. Interestingly, an increase in Mighty mRNA precedes that of MyoD during differentiation and overexpression of Mighty promotes differentiation in C2C12 muscle cells [15]. Furthermore, Mighty has recently been identified as a potential regulator of satellite cell chemotaxis during muscle regeneration [16]. Together, these data suggest that Mighty is a key developmental mediator of the growth effects of myostatin.

The effect of Mighty on satellite cell function and muscle regeneration is reminiscent of the interplay between TGFβ and Notch signaling [17]. In the satellite cells of older individuals, there is an increase in TGFβ/Smad pathway and a decrease in Notch signaling [18]. Activating Notch signaling in these cells resulted in the downregulation of cyclin-dependent kinase inhibitors concomitant with a decrease in the activity of TGFbeta as measured by Smad transcriptional activity [18]; this suggests that Notch can functionally inhibit TGFβ signaling and promote satellite cell proliferation. Furthermore, loading increases Notch activity [19], and an increase in the Notch family member delta-like 1 (DLK1) is responsible for the hypertrophic phenotype in the callipyge sheep [20]. Together, these data suggest that Notch may be involved in the regulation of myostatin/TGFβ signaling and skeletal muscle mass.

Notch activation is a complex process involving proteolytic cleavage of this single pass transmembrane receptor resulting in the production of the soluble Notch Intracellular Domain (NICD). NICD translocates to the nucleus where it can interact with transcription factors such as CSL (CBF-1, supressor of hairless, lag2) to create a transcriptional activation complex to increase the transcription of Notch target genes such as Hes-1 [21] or inhibit the expression of genes regulated by TGFβ [18] or activator protein-1 [22]. Since Mighty is a direct transcriptional target of myostatin, we hypothesized that the expression of Mighty mRNA could be used as a tool for determining the overall activity of the myostatin pathway in skeletal muscle following resistance exercise and in response to changes in Notch activity. The finding that Mighty expression increased in proportion to muscle growth led us to investigate the canonical myostatin signaling pathway and the activity of Notch in an attempt to understand how myostatin transcription is regulated following acute resistance exercise. Our data suggest that activation of Notch, as measured by the increase in NCID levels following loading, leads to repression of the myostatin pathway leading to increased Mighty expression.

## Materials and Methods

### Materials

The antibodies used in the study were: rabbit anti-phospho-Smad2 (#3108) and rabbit anti-GAPDH (#5174 lot#3) from Cell Signaling (Beverly, MA); total-Smad2/3 (#3862 lot#0509010605) from Chemicon (Billerica, MA); rabbit anti- Notch intracellular domain (#8925 lot#888593) from Abcam (Cambridge, MA); and mouse anti-SKI (#33693 lot#3007) from Santa Cruz Biotechnologies (Santa Cruz, CA). All other chemicals were from Sigma-Aldrich unless stated otherwise.

### Animals and Acute Resistance Exercise

All procedures were approved by the University of Dundee research ethics committee and performed under UK Home Office project license number 60/3441. Adult female Wistar rats weighing ∼200 g were obtained from Charles River Laboratories (Tranent, UK). All surgical and collection procedures on animals took place under inhaled anaesthetic using a 2.5% concentration of isoflurane throughout the procedure. The resistance exercise model was performed as described previously [12]. Briefly, the stimulation protocol consisted of two second tetanic contractions followed by 10 s recovery. After the sixth repetition a 1 min recovery was allowed. The protocol finished after the 10^th^ set and then immediately or 0.5, 3, 6, 18, and 48 hours following the acute bout of contractions, stimulated and contralateral muscles were rapidly removed, snap frozen in liquid nitrogen and stored at −80°C until processed.

### mRNA Extraction

mRNA was extracted from powdered muscles following homogenization in 1 mL of Trizol. The homogenate was centrifuged at 4°C for 10 minutes at 11000 rpm to remove insoluble material and RNA was then precipitated using chloroform separation, followed by isopropanol precipitation of the aqueous layer. RNA was quantified using a NanoDrop 1000 spectrophotometer (Thermo Scientific, Leicestershire, UK) at 260 and 280 nm.

### Reverse Transcription and qPCR

First strand cDNA was synthesized from 1 µg of RNA using the reverse transcription system (Promega, Hampshire, UK). RNA was denatured with oligo (DT) primers at 70°C for 10 minutes and the reverse transcription reaction was performed at 50°C for 1 hour. cDNA was diluted 1∶10 before use in qPCR.

qPCR was performed using an Eppendorf Mastercycler ep Real Time Thermocycler (Eppendorf) along with a SYBR Green Jump Start Taq ready mix (Sigma) and 96 well plates (Fisher). PCR reactions were performed according to manufacturer’s instructions with primers (sequence available at FMBlab.com) used at a concentration of 10 pM. PCR conditions were as follows: initial denaturation at 95°C for 15 minutes followed by 40 cycles of denaturation at 94°C for 15 seconds, annealing at 56°C for 30 seconds and extension at 72°C for 30 seconds. A melt curve was then performed to assess the specificity of the primers and the PCR product was run on a 1% agarose gel to check product purity. PCR efficiencies were also determined for each primer set. The ratio of expression of the gene of interest normalized against GAPDH was computed according to the equation proposed by Pfaffl [23].

### Correlation between Mighty and Growth

A correlation between the increase in Mighty mRNA 6 hours following resistance exercise and the growth of muscle following 6 weeks of training was performed by comparing the mean increase in Mighty mRNA in the current study to our historical data on muscle growth following stimulation two days per week for 6 weeks [12]. To facilitate the comparison, the current study used the identical stimulation model as well as rats of the same strain, age, and body weight.

### Western Blotting

Muscles were powdered on dry ice using a mortar and pestle and polytron homogenized in 10-fold mass excess of ice cold sucrose lysis buffer (50 mM Tris pH7.5, 250 mM Sucrose, 1 mM EDTA, 1 mM EGTA, 1% Triton X 100, 50 mM NaF, 1 mM NaVO_4_ Na_2_(PO_4_)_2_ and 0.1% DTT). This was vortexed for 30 minutes at 4°C and centrifuged at 4°C for 10 minutes at 10,000×g to remove insoluble material. Protein concentrations were determined using the DC protein assay (Bio-Rad, Hercules; California, USA).

Equal aliquots of protein were diluted in Laemmli sample buffer and boiled for 5 minutes. 5–10 µg of sample was then subjected to SDS-PAGE on 10% acrylamide gels at a constant current equal to 20 mA per gel and transferred to Protran nitrocellulose membrane (Whatman; Dassel, Germany) using a BioRad semidry transfer apparatus at 100 V for 1 hour. Membranes were blocked in 5% dry milk in TBST (Tris-buffered saline +0.1% Tween), and then incubated over night at 4°C with appropriate primary antibody in TBST at 1∶1,000. The membranes were then washed 3x in TBST before incubation for 1 hour at room temperature with peroxidase-conjugated secondary antibodies in TBST at 1∶10,000 (Perbio Science; Cramlington, UK). Antibody binding was detected using an enhanced chemiluminescence HRP substrate detection kit (Millipore; Watford, UK). Imaging and band quantification were carried out using a Chemi Genius Bioimaging Gel Doc System (Syngene; Cambridge, UK).

### Electroporation

Electroporation was carried out using an ECM 830 Square Wave Electroporation System (Harvard Apparatus, Holliston, MA). Briefly, 6 µl of control or experimental plasmid DNA dissolved in sterile PBS at a concentration of 2.5 µg/µl was injected at the proximal and distal ends of the surgically exposed tibialis muscle (TA). The control DNA, pDsRed2-C1 (Clontech, Mountain View, CA), contained the dsRed cDNA driven by the cytomegalovirus (CMV) promoter. The experimental DNA contained the 2.39 kB of human Notch-1 generously donated by Professor Tom Kadesch, University of Pennsylvania School of Medicine, which comprises the intracellular (transcriptionally active) domain of the protein driven by the CMV promoter [24]. Both right and left legs were electroporated and the order of the injections was randomized. Following injection, the muscles were electroporated with 8×20 ms pulses at 160 V/cm. The skin was closed, the animals were given an analgesic injection of buprenex (buprenorphine hydrochloride) and allowed to recover. One week following electroporation, muscles were removed, frozen in liquid nitrogen and RNA was isolated as described above and qPCR was performed using primers to human NICD (to detect NICD expression from the injected plasmid), the canonical Notch target Hes-1 (hairy and enhancer of split-1), and Mighty.

### Statistical Analysis

One-way analysis of variance ANOVA (BrightStat.com) and Tukey honestly significant difference posthoc test were used to determine differences in protein level/phosphorylation and mRNA expression. Values are displayed as mean ± SEM, with statistical significance set at 0.05.

## Results

### Effect of Acute Resistance Exercise on Myostatin Transcriptional Activity

**Figure 1:**
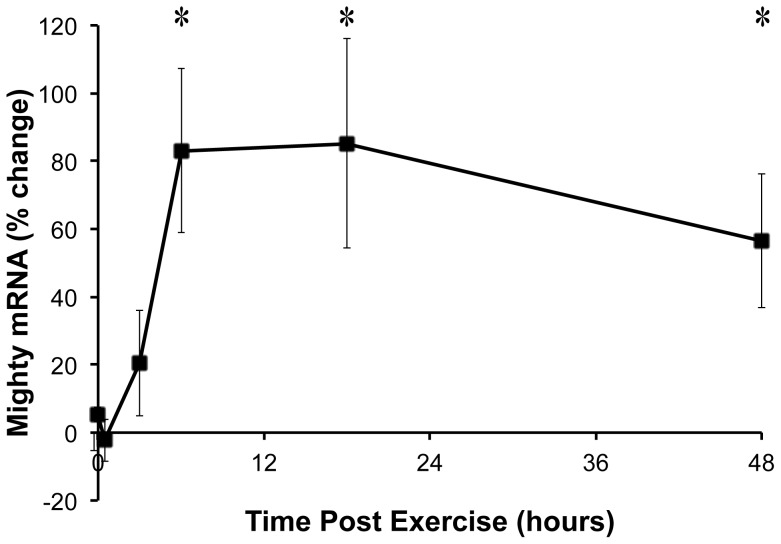
Mighty expression in TA muscle following resistance exercise. The expression of Mighty was determined at the end of a 20 min bout of high force lengthening contractions and then 0.5, 3, 6, 18, and 48 hours later. Mighty mRNA increased following resistance exercise starting at 6 h. *indicates significantly greater than the contralateral control leg (n = 4, p<0.05).

**Figure 2:**
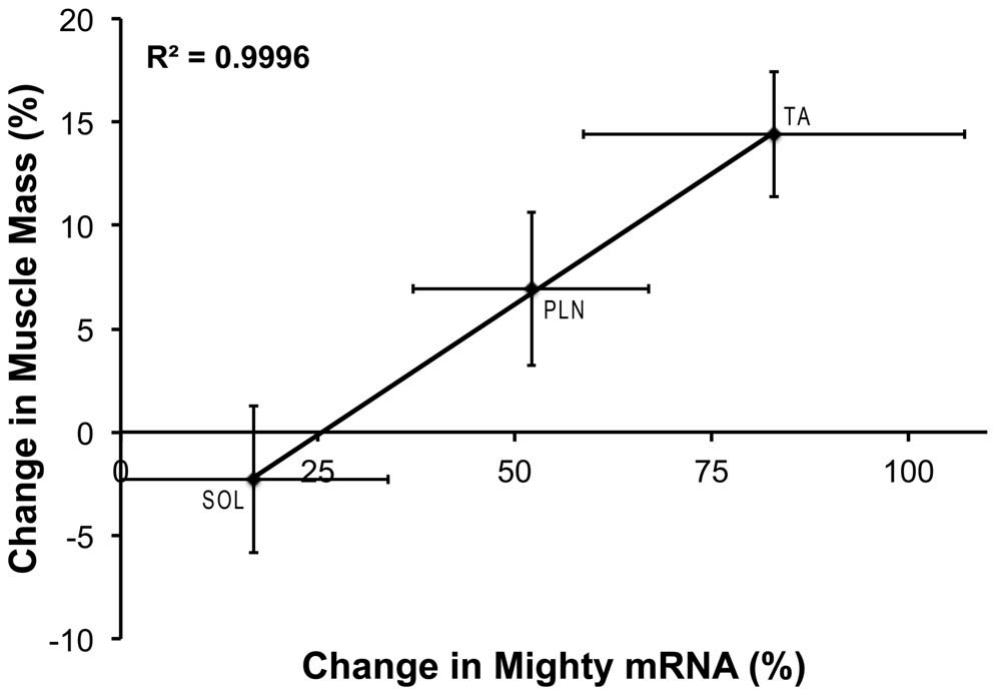
Correlation between average Mighty mRNA expression in 3 different muscles 6 hours after stimulation and average hypertrophy after 6 weeks of training. Since the expression of Mighty increased 6 h following an acute bout of resistance exercise in the TA muscle, Mighty expression was determined in the soleus (SOL) and plantaris (PLN) muscles as well. Mighty mRNA at 6 h was plotted against data on the increase in muscle mass following 6 weeks of repeated bouts of resistance exercise from a previous study using this model [12]. There was a significant correlation between the mean difference in Mighty mRNA in each muscle at 6 h and the mean difference in muscle mass following 6 weeks of training (R^2^ = 0.9996).

The expression of the myostatin target Mighty was determined immediately following, 0.5, 3, 6, 18, and 48 hours after resistance exercise. From 6 to 48 hours following resistance exercise, Mighty expression was increased (Figure 1), suggesting myostatin transcriptional activity decreased in the active muscle following acute resistance exercise. At 48 hours, the amount of Mighty decreased towards baseline. We next decided to exploit the differential growth response of the muscles stimulated with this model [25], [26]. The muscles in the anterior compartment of the lower limb contract eccentrically and grow to a greater extent than the muscles in the posterior compartment allowing for comparisons to be made between the differential growth response of a particular muscle and the change in gene expression in that muscle. Comparing the expression of Mighty in the different hindlimb muscles at the 6-hour time point with previous data on the growth of individual muscles over 6 weeks [12] revealed a strong correlation (R^2^ = 0.9996; Figure 2). The relationship between Mighty expression and skeletal muscle hypertrophy suggests that increasing Mighty mRNA (inhibiting myostatin transcriptional activity) may be important in the acute hypertrophic process.

### Effect of Acute Resistance Exercise on Canonical Myostatin Signaling

**Figure 3:**
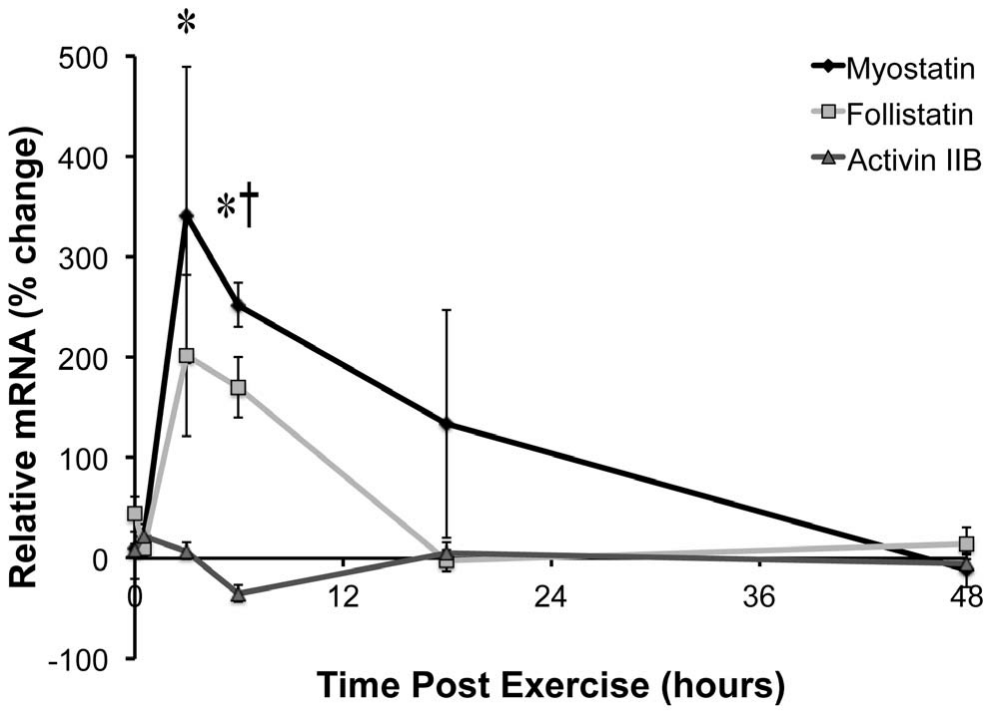
Canonical myostatin signaling following resistance exercise. The expression of genes within the myostatin pathway was determined in TA muscle at the end of a 20 min bout of high force lengthening contractions and then 0.5, 3, 6, 18, and 48 hours later. Myostatin and follistatin mRNA increased following resistance exercise starting at 3 h. The expression of the myostatin receptor (ActRIIB) was unchanged at any time point. *indicates significantly greater than the contralateral control leg for follistatin and † indicates significantly greater than the contralateral control leg for myostatin (n = 4, p<0.05).

**Figure 4:**
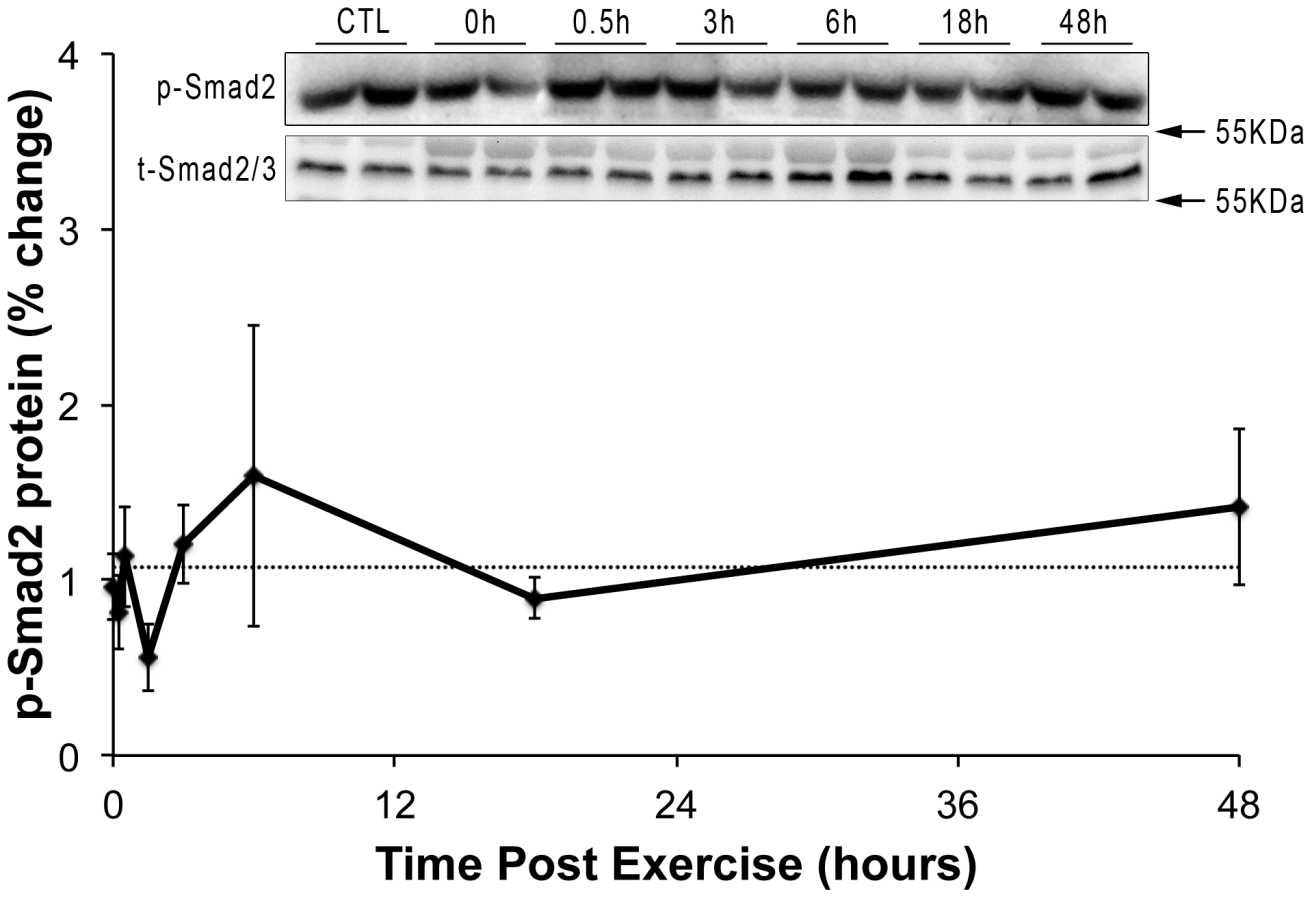
Smad2 phosphorylation following resistance exercise. The degree of Smad2 phosphorylation was determined in an attempt to measure myostatin signaling *in vivo* at the end of a 20 min bout of high force lengthening contractions and then 0.5, 3, 6, 18, and 48 hours later. Smad2 phosphorylation was unchanged at any time point following resistance exercise (n = 4).

Since Mighty expression is inversely related to myostatin/Smad signaling [15], we next sought to determine whether changes in myostatin and Smad phosphorlylation triggered the increase Mighty transcription. The expression of myostatin and its inhibitor follistatin were both increased following resistance exercise (Figure 3) with follistatin significantly increased at 3 and 6 hours (Control = 1.4±18.6%; 3 h = 201.5±80.5%; and 6 h = 170.3±30.1%) and myostatin increased at the 6 h time point (Control = 28.1±1.99%; 6 h = 252.1±21.8%). The expression of the ActRIIB gene remained unchanged throughout the time course (Figure 3). Consistent with both myostatin and follistatin increasing similarly following acute resistance exercise, Smad2 phosphorylation did not change at any point after acute resistance exercise (Figure 4).

### Notch Activation and Myostatin Signaling

**Figure 5:**
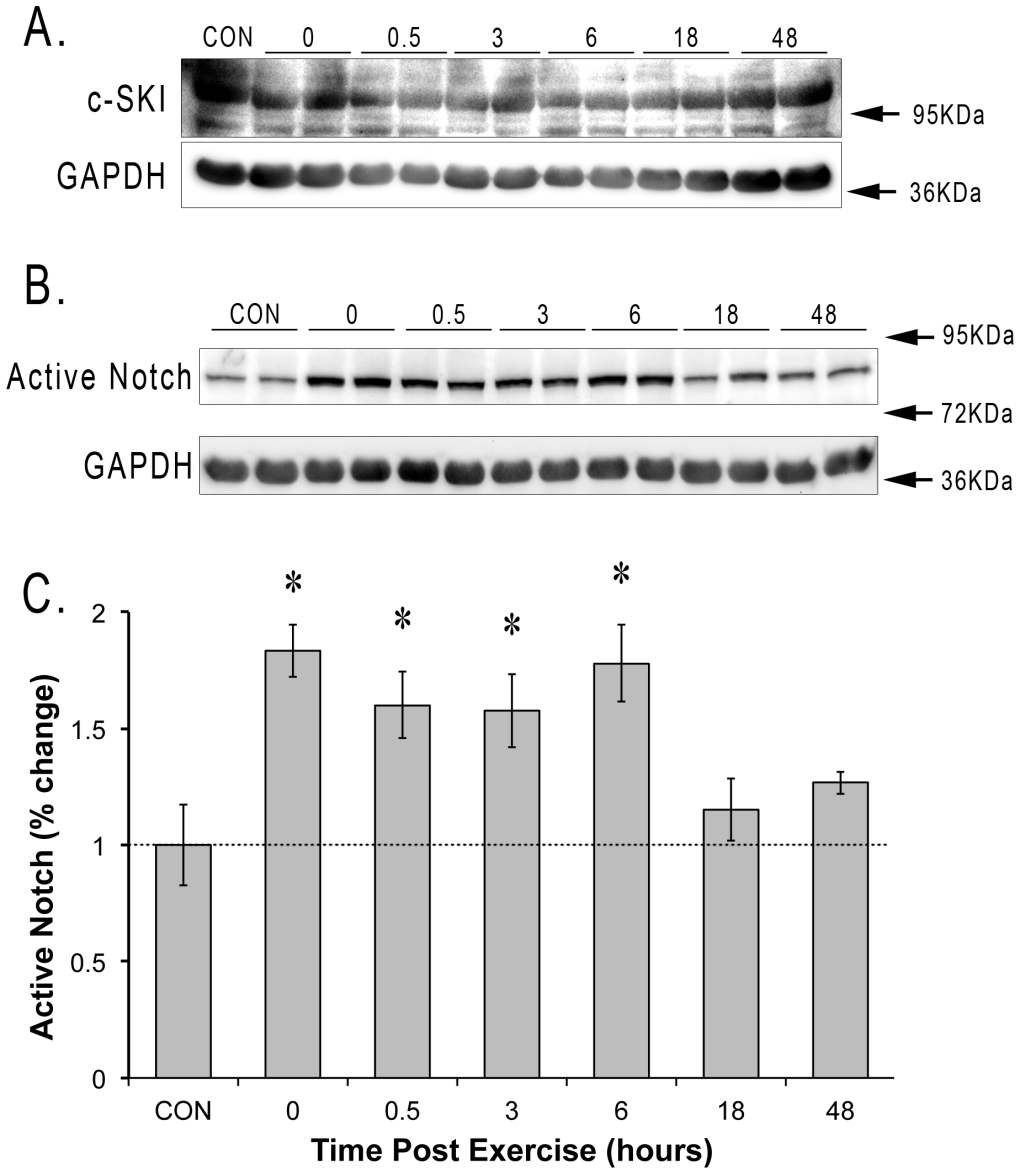
TGFβ/Smad transcriptional inhibitors following acute resistance exercise. The levels of the Smad inhibitors SKI and active Notch were determined at the end of a 20 min bout of high force lengthening contractions and then 0.5, 3, 6, 18, and 48 hours later. SKI and Notch protein was normalized to GAPDH. GAPDH levels were unchanged in the 48 hours after acute stimulation. *indicates significantly increased versus control (n = 4, p<0.05).

Since there was no relationship between myostatin, Smad2 phosphorylation, and myostatin transcriptional activity, we determined the levels of the transcriptional repressors SKI and activated Notch. The levels of SKI protein were unchanged throughout the 48 h following resistance exercise (Figure 5A). However, immediately after resistance exercise we noted an 83±11.2% increase in the amount of activated Notch (Figure 5B). The activated Notch remained high for 6 hours (78.1±16.6%) before returning to control levels by 18 hours (Control = 1.0±1.7%; 18 h = 15±13%).

### NICD and Hes-1 and Mighty Expression

**Figure 6:**
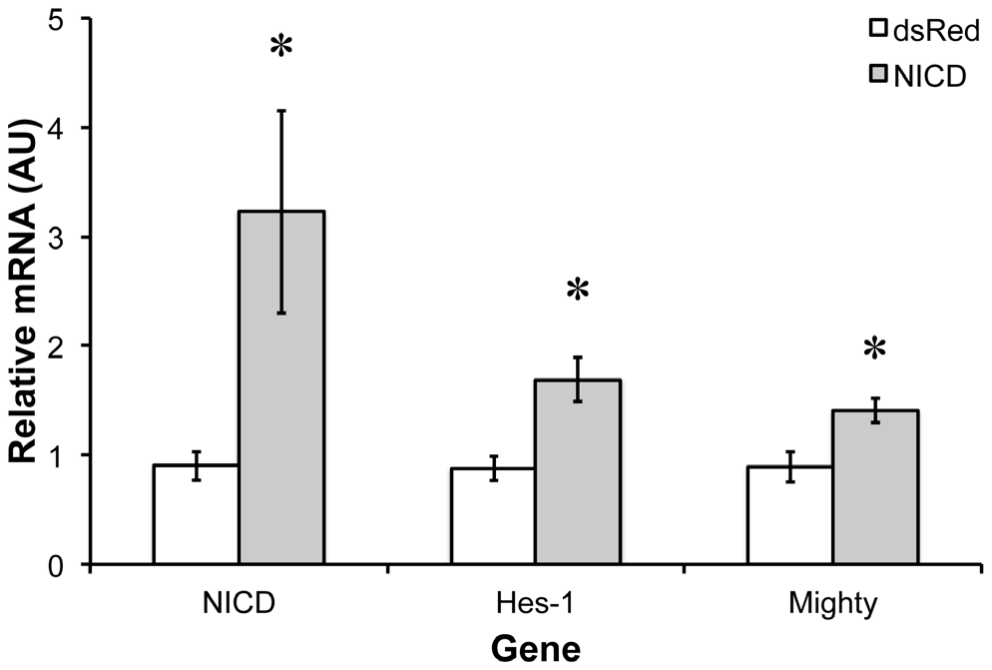
NICD (Notch Intracellular Domain) electroporation increases canonical Notch signaling and Mighty expression. The expression of the NICD, Hes-1, and Mighty was quantified one week following electroporation of the human NICD into the tibialis anterior muscle of mice. Expression of each gene was normalized to GAPDH. GAPDH levels were unchanged following electroporation. *indicates significantly increased versus control (n = 4, p<0.05).

To determine whether increased active Notch associates with increases in Mighty expression, control (dsRED, empty plamid) or NICD (plasmid containing the human Notch-1 intracellular domain cDNA) cDNA was electroporated into the tibialis anterior muscle of wild type C57/Bl6 mice (Figure 6). One week following injection, the whole muscle level of the human NICD increased 345±53%. The canonical Notch target Hes-1 increased 94.4±7.32%. Mighty mRNA increased an equivalent amount (63±13.4%), indicating that overexpression of NICD increases Notch target gene expression and Mighty mRNA.

## Discussion

Following resistance exercise, there is a small but significant increase in the expression of the myostatin target gene Mighty that correlates with the increase in muscle mass following training. This is the first time that a relationship between acute myostatin signaling and skeletal muscle hypertrophy has been observed, suggesting that inhibition of myostatin may play a role in load-induced muscle hypertrophy. Since myostatin inhibits Mighty expression, this suggests that myostatin transcriptional activity may be diminished from 6–48 hours following resistance exercise. The decrease in myostatin transcriptional activity was not correlated with changes in myostatin or follistatin mRNA or Smad2 phosphorylation. Instead, this decrease in transcriptional activity was related to an increase in the cleavage and activation of Notch, which is known to interact with Smad2/3 [27]. Electroporating a plasmid containin the human NICD cDNA into muscle resulted in an increase in both the canonical Notch target Hes-1 and the myostatin target Mighty, suggesting that resistance exercise results in the cleavage and activation of Notch. Notch would then translocate to the nucleus and decrease myostatin/Smad transcriptional activity.

Mighty was cloned in a subtractive hybridization screen comparing myostatin knockout and wild type control mice [15]. The cloned gene was expressed during myoblast differentiation and overexpression of Mighty promoted muscle cell differentiation. When virally expressed in *mdx* muscle, Mighty increased muscle fiber size and overall muscle mass, suggesting that Mighty could potentially play a role in skeletal muscle hypertrophy [15]. Importantly, acute treatment of muscle cells with myostatin decreased Mighty promoter activity in a dose-dependent manner that was dependent on activation of Smad2, suggesting that Mighty mRNA levels would be a good marker for the activity of the myostatin pathway [15]. The current report is the first report showing that Mighty levels are increased following acute resistance exercise and the correlation between Mighty mRNA and hypertrophy supports a role for this gene in muscle growth. However, how Mighty affects muscle hypertrophy has yet to be determined.

Even though a number of reports have shown that myostatin is transcriptionally downregulated by acute resistance exercise in rats [9], [10], we observed an increase in myostatin mRNA within the working muscle in the hours following acute resistance exercise. Our finding is in contrast to the decrease in myostatin mRNA that occurs with overload hypertrophy [28], [29], [30]. Furthermore, when other researchers have measured myostatin mRNA in rats after 4 days of electrical stimulation, they noted a decrease [31]. One possible explanation for these differences is that Garma and colleagues quantified myostatin relative to 18S RNA and not GAPDH as we did in this study. This is relevant since these same authors measured a 30% increase in total RNA. Since ribosomal RNA makes up ∼80% of total RNA, 18S rRNA levels would increase disproportionately, making myostatin mRNA appear to go down. Interestingly, in spite of the rise in myostatin mRNA in the current study, Smad2 phosphorylation was unchanged following acute resistance exercise possibly due to an almost identical increase in follistatin mRNA. This demonstrates the complexity of myostatin signaling and shows that measuring the mRNA, or even the protein levels, of a single component of the myostatin pathway may not provide a reliable estimate of the transcriptional activity of the pathway.

Carlson et al. [18] have demonstrated that Notch can directly inhibit TGFβ/Smad signaling in skeletal muscle cells. These authors suggested that an imbalance between TGFβ/Smad and Notch signaling may underlie the inability of skeletal muscle to regenerate in older animals and that this may lead to sarcopenia [32]. Interestingly, both myostatin inhibition and increasing Mighty induce satellite cell proliferation [16], [33], [34], [35], suggesting that the positive effects of TGFβ inhibition are potentially mediated through Mighty. However, even though Mighty may play a role in the activation of satellite cells, whether this is the role of Mighty following resistance exercise has yet to be determined. Interestingly, the Notch family member DLK1 can induce skeletal muscle hypertrophy [20] and testosterone increases active Notch in old muscles as part of the hypertrophic response [32]. Since satellite cell activation can be accelerated by testosterone [36] and testosterone can activate Notch and inhibit TGF-β, [32], [37], this suggests that Notch/Mighty signaling is important in load-induced skeletal muscle growth, possibly through the modulation of satellite cell activation. Consistent with this hypothesis, we show here that active Notch is increased immediately following resistance exercise and remains elevated for at least 6 hours. This time course of activation fits well with the increase in Mighty mRNA. Furthermore, electroporation of the NICD into skeletal muscle results in an increase in Mighty gene expression suggesting that notch cleavage and activation can block myostatin transcriptional activity *in vivo*.

In summary, acute resistance exercise results in a decrease in the transcriptional activity of myostatin and therefore an increase in the expression of Mighty, a gene that is associated with increases in skeletal muscle mass. The increase in Mighty expression following resistance exercise correlates with muscle growth, but is not the result of a decrease in canonical myostatin signaling. Instead, acute resistance exercise results in an increase in active Notch, a known inhibitor of TGFβ/Smad transcription. Lastly, overexpression of active Notch in skeletal muscle is associated with a down regulation in myostatin transcriptional activity as estimated by increased Mighty gene expression. These data indicate that inhibition of myostatin transcriptional activity by Notch may be important in load-induced skeletal muscle hypertrophy and identify Mighty as a genetic readout of myostatin signaling.

.
